# Temperature changes among organophosphate poisoned patients, Tehran- Iran

**DOI:** 10.1186/2008-2231-20-52

**Published:** 2012-10-15

**Authors:** Haleh Talaie, Hamid Owliaey, Abdolkarim Pajoumand, Mona Gholaminejad, Omid Mehrpour

**Affiliations:** 1 Toxicological Research Center, Loghman-Hakim Hospital, Shahid Beheshti University of Medical Sciences, South Karegar Avenue, Tehran, 1333631152, Iran; 2 Poison Center, Loghman-Hakim Hospital, Shahid Beheshti University of Medical Sciences, South Karegar Avenue, Tehran, 1333631152, Iran; 3 Medical Toxicology and Drug Abuse Research Center (MTDRC), Pasdaran Avenue, Birjand University of Medical Sciences, Birjand, 9713643138, Iran; 4 Department of Clinical Toxicology and Forensic Medicine, Faculty of Medicine, Birjand University of Medical Sciences (BUMS), Ghaffari Avenue, Birjand, 9717853577, Iran

**Keywords:** Organophosphate, Pesticide, Poisoning, Tympanic temperature

## Abstract

**Background:**

Acute poisoning with organophosphorus compounds (OPs) is a major global clinical problem in the developing countries. There have been many animal studies and few human surveys on the effects of organophosphorus pesticide (OP) poisoning on thermoregulation. The aim of this prospective study was to document the pattern of tympanic temperature changes among OP poisoned patients throughout the length of their hospital stay.

**Methods:**

60 patients with diagnose of organophosphate poisoning were included in this study. Questioner was filled out by trained nurses including demographic, clinical and paraclinical data. Tympanic temperature and Pulse rate data of the cases were collected on five- occasions after admission.

**Results:**

There were 41 (68.3%) male and 19 (31.7%) female, with a mean age of 34.4 ±19.4 years (range 13–89 years). Forty five patients had intentional poisoning for suicidal attempt. At the time of entry, the mean tympanic temperature, pulse rate, respiratory rate and blood pressure (systolic and diastolic) of the OP poisoned patients were respectively 37.1+/−0.6°C (36.0- 39.5), 91+/−18 (55–145), 18+/−5.6 (8–44), 116+/−20 mm Hg (70–170) and 75+/−11.6 mm Hg (40–110). 41.7% of the cases had serum butyryl cholinesterase activities (BChE) ≥ 50% normal (≥1600 mU/ml). Our patients had normal temperature at the time entry (mean = 37.1). Tympanic temperature decreasing below 36°C was not detected among the patients during the study period. A rise in mean tympanic temperature was found after atropine administration.

**Conclusion:**

Our study showed hypothermia was not considerable factor among organophosphate poisoned patients, although more studies with similar situations in tropical countries are needed.

## Introduction

Acute poisoning with organophosphorus compounds (OPs) is a major global clinical problem in the developing countries with a significant cause of morbidity and mortality. It can occur in a variety of situations such as agricultural use, accidental exposure, suicide and, rarely, homicide [1,2]. Organophosphorus compounds form a large family of ~50 000 chemical agents [3].

These compounds act by inhibiting acetylcholinesterase activity at muscarinic and nicotinic receptors in the brain and different parts of neuromuscular junctions. Four clinical syndromes that have been described in patients with OPs poisoning, are cholinergic crisis, intermediate syndrome, delayed neuropathy and chronic organophosphate inducted neuropsychiatric disorder. Whichever stages has special signs and symptoms [4].

The conventional and standard treatment involves supportive care, detoxification and administration of intravenous atropine sulfate, a central and peripheral muscarinic receptor antagonist, and pralidoxime chloride to counter acetyl cholinesterase inhibition at the synapse [4-6].

However, the pathophysiology of OPs poisoning is not completely known, reports indicate that OPs interfere with the control of acetylcholine-regulated homeostatic mechanisms such as temperature regulation. Studies on laboratory rodents showed hypothermia induced by direct CNS administration of cholinergic agonists in the region of their hypothalamus or cerebral ventricles, on the contrary other survey found heat production. It is suggested that temperature's variables may be dose dependent, for example, generally higher doses were associated with hypothermia, and hyperthermia was only seen with lower doses [6,7].

Other studies indicated that a period of hypothermia followed by a fever of delayed onset in OP poisoning, without existence of viral or bacterial infection [8-11].

According to several studies on human OP poisoning, elevation in body temperature was a frequent outcome, but cases were complicated by concurrent illnesses (in particular aspiration pneumonitis/pneumonia) and interventions that may of themselves produce high temperatures, in particular anticholinergic agents [6,12]. The aim of this study was to obtain the pattern of tympanic temperature changes among OP poisoned patients throughout the length of their hospital stay.

## Methods

This prospective chart review study was conducted on patients with OP poisoned suspicious that admitted to our 18- bed Toxicological Intensive Care Unit (TICU) and 45- bed poison ward of Loghman Hakim Hospital Poison Center (LHHPC), from October 2010 to September 2011. This hospital is a unique care teaching and referral poison treatment center in Tehran with nearly an annual average of 20000 hospital visits [13]. The study protocol with code number 101 was reviewed and approved by ethics review committee in Research Deputy Department of the Shahid Beheshti University of Medical Sciences, Tehran, Iran. During the period of study, sixty cases with diagnosis of OP poisoning included. The diagnose was confirmed by based on information taken either from the patient or from the Patient’s family about the OP exposure, the odor of OPs in the gastric contents and cholinergic clinical features and measuring butyrylcholinesterase activities (BChE) in the first 24 hours. The serum BChE categorized in 3 groups which included: ≤20% normal, between 20 to 50% and ≥50% normal. Standard treatment was used for patients: gastric lavage, whole body surface washing, activated charcoal administration (1 g/kg by nasogastric tube), intravenous atropine and pralidoxime in OP poisoned patients, and supportive measures such as mechanical ventilation (if necessary). Questioner was filled out by trained nurses including demographic, clinical and paraclinical data. The patients' demographic information (sex, age), vital signs at the entrance, co ingestion with other drugs or substances, clinical manifestation (muscarinic and nicotinic symptoms), CNS status, intubation, acetyl cholinesterase levels, dose of atropine and paralidoxims (bolus administration, infusion) and the length of administrate of these drugs were extracted by review of medical records. The Glasgow Coma Scale (GCS) of patients were determined. Tympanic temperature and Pulse rate data of the cases were collected in five- occasions. There were included: at entry time, after atropine treatment, after 24 hrs, after 72 hrs and at the discharge. More data such as aspiration pneumonia complication, opioid's withdrawal syndrome and other drugs' medication mentioned. The statistical analysis was performed with Statistical Product and Service Solutions (SPSS) version 16 (SPSS Inc., Chicago, IL, USA). Data of the participants were analyzed through appropriate statistical testes, such as Chi-square test (*χ*2) for categorical and Student's *t*-test. P-values equal to or less than 0.05 considered significant.

## Results

Over the 1- year, 60 poisoned cases with OP poisoning were registered in Loghman Hakim Hospital, with a mean age of 34.4 ±19.4 years (Range: 2–89 years). There were 41 (68.3%) male and 19 (31.7%) female.

Table 1 showed distribution of age and sex among patients. Forty five patients (75%) had intentional poisoning for suicidal attempt and 15 cases (25%) had accidental exposure. Four patients had positive history of co ingestion with other drugs or substances which was ethanol (n = 2) and benzodiazepine (n = 2).

**Table 1:** Distribution of age and sex among patients

**Age**	**1-10y**	**11-20y**	**21-30y**	**31-40y**	**41-50y**	**51-60y**	**61-70y**	**71-80y**	**81-90y**	**Total**
**Sex**	**N (%)**	**N (%)**	**N (%)**	**N (%)**	**N (%)**	**N (%)**	**N (%)**	**N (%)**	**N (%)**	**N (%)**
**Male**	-	4 (9.8)	18 (43.9)	4 (9.8)	5 (12.2)	4 (9.8)	2 (4.9)	2 (4.9)	2 (4.9)	41 (100)
**Female**	1 (5.3)	6 (31.6)	9 (47.4)	1 (5.3)	1 (5.3)	-	-	1 (5.3)	-	19 (100)
**Total**	1 (1.7)	10 (16.7)	27 (45)	5 (8.3)	6 (10)	4 (6.7)	2 (3.3)	3 (5)	2 (3.3)	60 (100)

Most of the patients (n = 46) arrived to the hospital during the first 6 hours of ingestion (Table 2).

**Table 2:** Time interval between exposure and hospital arrival (h)

**Time to admission (h)**	**unknown**	**<1 (h)**	**1-6(h)**	**7-24(h)**	**>24(h)**	**Total**
	**N (%)**	**N (%)**	**N (%)**	**N (%)**	**N (%)**	**N (%)**
**Organophosphorus**	7 (11.7)	13 (21.7)	33 (55.1)	3 (5.1)	4 (6.7)	60 (100)

GCS of 56 patients during the first 24 hours were above 10 and 4 case had GCS between 3–6. At the time of entry, the mean tympanic temperature, pulse rate, respiratory rate and blood pressure (systolic and diastolic) of the OP poisoned patients were respectively 37.1+/−0.6°C (36.0- 39.5), 91+/−18 (55–145), 18+/−5.6 (8–44), 116+/−20 mm Hg (70–170) and 75+/−11.6 mm Hg (40–110). The most frequent clinical signs and symptoms were miosis and hyper salivation followed by vomiting (Table 3).

**Table 3:** Clinical signs and symptoms of the OP poisoned patients

**Muscarinic Patient Signs and Symptom**	**Number (%)**	**Nicotinic Patient Signs and Symptom**	**Number (%)**
**Hyper salivation**	22 (36.7)	**Fasciculation**	3 (5)
**Tearing**	15 (25)		
**Miosis**	22 (36.7)		
**Vomiting**	19 (31.7)	**Muscle Weakness**	7 (11.7)
**Diarrhea**	12 (20)		
**Sweating**	11 (18.3)		
**Bradycardia**	7 (11.7)		
**Abnormal Lung Auscultation (Rales , wheeze, Ronchy)**	22 (36.5)		

41.7% of the cases had serum butyryl cholinesterase activities (BChE) ≥ 50% normal (≥1600 mU/ml). Patients' serum butyrylcholinesterase activities (BChE) in the first 24 hours were shown in Table 4.

**Table 4:** OP poisoned patients' serum butyrylcholinesterase activities (BChE) in the first 24 hours

**Serum BChE Patient**	**≤20% Normal (≤640** **mU/ml)**	**Between 20 to 50 % Normal (640 < & <1600** **mU/ml)**	**≥50% Normal(≥1600 mU/ml)**	**Total**
**Number (%)**	19 (31.7)	16 (26.7)	25 (41.7)	60 (100)

The average bolus atropine dose in OP poisoned patients' records was 7.4 ± 16.9 mg and the highest dose was 100 mg just in one case. Atropine was given during 5.5 ± 3.6 days (range 4 hours - 15 days) and the average infusion atropine dose was 110.2 ± 90.7 mg.

A total of 31 (51.7%) patients received pralidoxime. The mean bolus paralidoxime dose was1.4 ± 0.6 g. Pralidoxime was administered for 6.3 ± 5 days (Range: 2 hours -18 days) and the mean dose was 41.6 ± 30 g.

As other medication that we used, benzodiazepines (Diazepam–Midazolam) were the most frequent (Table 5).

**Table 5:** Other medications that were used among the OP poisoned patients

**Other Medications**	**Patients Number (%)**
NAC*	8 (13.33)
**Sodium bicarbonate**	14 (23.33)
**Benzodiazepines Diazepam Midazolam**	15 (25) 19 (31.66)
**Magnesium sulphate**	12 (20)

NAC: N acetyl cysteine.

**Figure 1:**
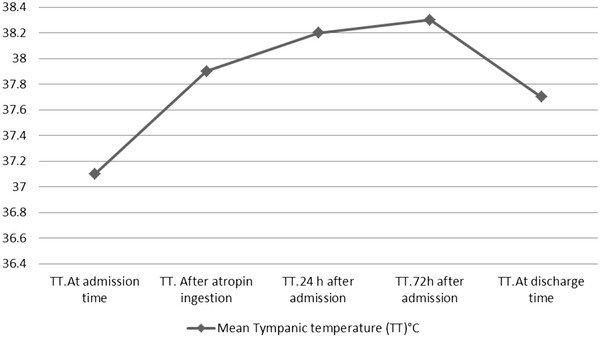
Mean tympanic temperature changes among patients throughout the length of their hospital stay.

**Figure 2:**
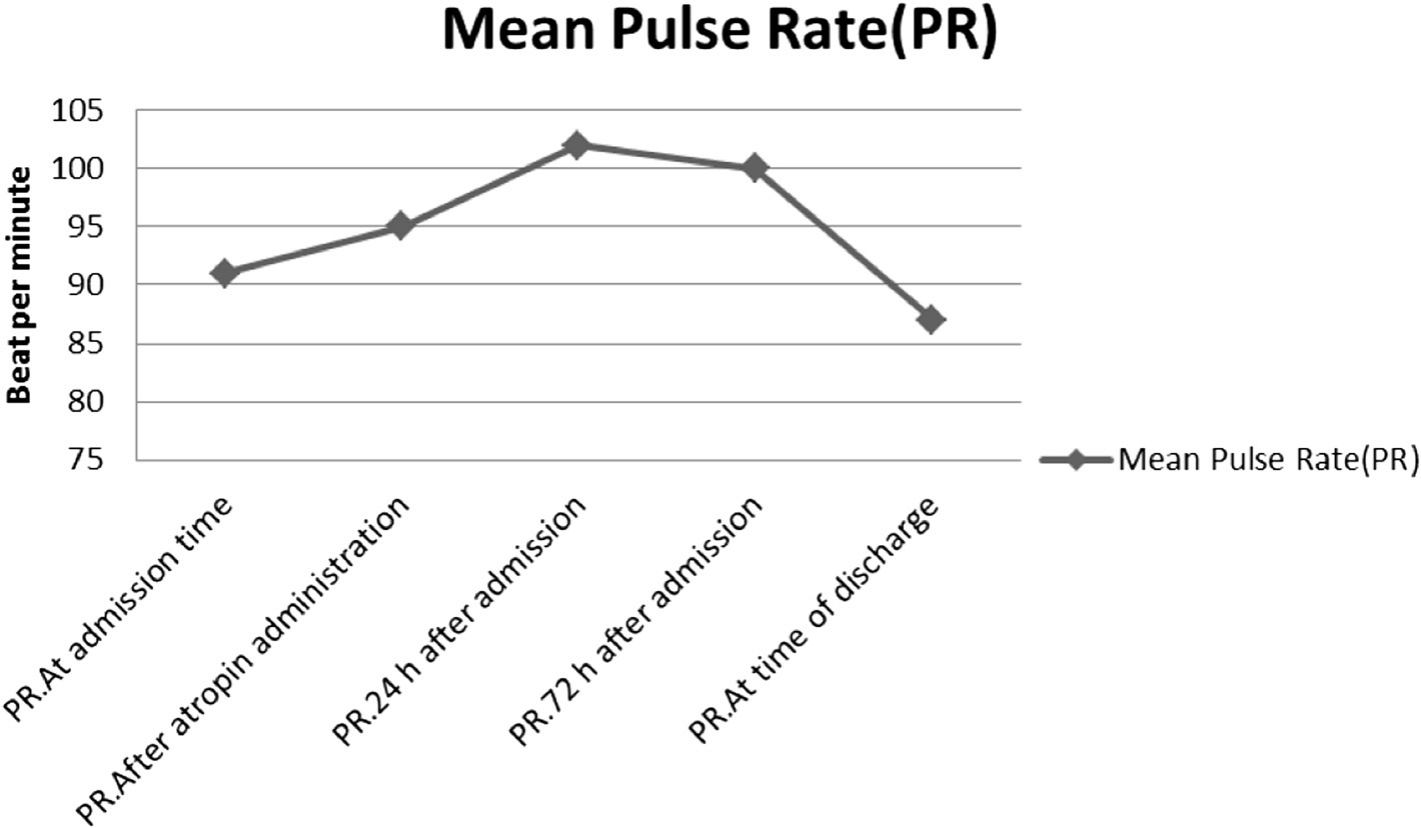
Mean pulse rate changes among patients throughout the length of their hospital stay.

Figure 1 showed the mean tympanic temperature changes among patients throughout the length of their hospital stay. Figure 2 showed the mean pulse rate changes among patients throughout the length of their hospital stay. Hypothermia was not detected among the patients even at the time entry. A rise in mean tympanic temperature was found after atropine administration.

Intubation and consequent mechanical ventilatory support was needed for 30 (50%) patients. It is notable that14of these mechanically ventilated patients were complicated by aspiration pneumonia (AP). Out of 14 patients with AP, 4 cases died. Seizure occurred in 6 patients. Drugs withdrawal was reported in 2 cases. Ten of the 60 patients died and mortality rate was 16.6%.

## Discussion

In this study we found that patients had mean tympanic temperature 37.1°C at the time entry and a rise in mean tympanic temperature after atropine administration.

OPs have been reported to interfere with temperature regulation [7]. Most of surveys about effect of organophosphate on temperature regulation are from animal studies. Direct CNS administration of cholinergic agonists in the hypothalamus or cerebral ventricles of rodents induced hypothermia, but other studies reporting heat production and fever [6,7]. This different may be related to dose of organophosphate; in general, higher doses of OPs were associated with hypothermia, and hyperthermia was seen with lower doses of OPs [6]. Other studies have been proved that without other classical signs of bacterial or viral infection, OP poisoning induced a period of hypothermia followed by a delayed onset of fever [8,9]. Moreover, hot and cold environments may changes sensitivity of rats to AChE inhibitors and also hypothermic and hyperthermic effects in animal models [6].

Very few study have evaluated effect of OP poisoning on temperature regulation, Moffatt A et al. in a prospective case series study found that OP poisoning causes an initial fall in body temperature, and this is followed by a period of normal to high body temperature, as measured by tympanic thermometry [6], which was similar to our study, although most of our cases had normal temperature at time of admission, it was important to know that in Moffatt et al. study, hypothermia may be related to co ingestion of alcohol in some patients.

Numerous animal surveys noted purely hypothermic or hyperthermic responses to OP poisoning and they suggested that there was a significant correlation between OP exposure and hypersensitivity to ambient temperatures [6,12,14,15]. On the other hand, we detected hyperthermia among the patients after atropinization.

Some studies noted that elevation in body temperature was a frequent outcome, but cases were complicated by concurrent illnesses (in particular aspiration pneumonitis/pneumonia) and interventions that may of themeselves produce high temperatures, in particular anticholinergic agents. They noted that it is unknown whether atropine was responsible for the temperature or whether high doses of atropine were given to patients with severe OP poisoning, and fever was a manifestation of severe OP poisoning [6,12,16,17].

OP poisoning is a common poisoning in the warmer regions of the world, such as South Asia, the Middle East and Africa. So, reported fever in patients with OP poisoning may represent a loss of normal thermoregulation [6].

The most important limitation of this study was the need to antidotes therapy that may affect temperature regulation. Moreover, the treatment is individualized to each patient; another limitation of study was co-ingestion of other agents in some cases which may affect on temperature regulation. Also temperatures may also have been affected by a patient’s location in hospital and temperature of surrounding environment the patient in each case.

## Conclusion

Hypothermia was not detected among the patients even at the time entry. A rise in mean tympanic temperature was found after atropine administration, although we need more study with similar situations in tropical countries.

## Competing interests

The authors declare that they have no competing interests.

## Authors’ contributions

HT and AP gave the idea and revising the manuscript critically for important intellectual content, HO collected data and MG did bibliography and drafted the article and OM completed/edited/revised the article. All authors read and approved the final manuscript.
